# Opportunities for Graduate Study in Special Hospitals

**Published:** 1913-09-06

**Authors:** 


### OPPORTUNITIES FOR GRADUATE STUDY IN SPECIAL HOSPITALS.

The London Post-Graduate Association issue tickets which admit to the clinical practice of the leading general and special hospitals in the metropolis. The fee for three months is £10 10s. The cards are issued on personal application between the hours of 10.30 and 1 (except on Saturdays), at the office of the Association, 20 Hanover Square, London, W. Evidence of qualification will be required. The cards are non-transferable, must be signed by the holder, and must be shown at the request of the authorities of the hospitals visited. A list of operations, clinical lectures, and other arrangements can be seen daily at the Secretary's office. Special classes for Post-graduate students only will be held during the forthcoming session at the Westminster Hospital, the Brompton Hospital for Consumption and Diseases of the Chest, the National Hospital for the Paralysed and Epileptic, St. Mark's Hospital for Diseases of the Rectum, and the Medical Graduates' College and Polyclinic. Holders of the Post-Graduate Association ticket are admitted to these classes on payment of the special fees.

### Ophthalmic Hospitals.

Ophthalmic hospitals are the Central London Ophthalmic Hospital, Judd Street, W.C.; the Royal London Ophthalmic Hospital, City Road, E.C.; the Royal Eye Hospital, St. George's Circus, S.E.; and the Royal Westminster Ophthalmic Hospital, King William Street, W.C. In each of these the fees charged are very modest.

### St. John's Hospital for Diseases of the Skin.

Out-patient Department, 49 Leicester Square; In-patient Department, 262 Uxbridge Road, W. This hospital has a well-equipped in-patient department, with 40 beds. It has a School of Dermatology at 49 Leicester Square, which is conducted by the medical staff of the hospital. During the winter the free course of Chesterfield lectures is given by Dr. Morgan Dockrell, and is always well attended by the profession. The out-patient department has recently been rebuilt, and contains a spacious laboratory and special electrical department. Special courses in the pathology and bacteriology of the skin may be arranged for. The daily clinics at 2, and every evening at 6 (except Saturday), are open to practitioners, upon signing the Visitors' Book. About 800 cases are seen every week.

### Queen Charlotte's Lying-in Hospital.

Qualified medical practitioners and medical students are admitted to the practice of this hospital. Last year (1912) there were 1880 women delivered in the wards, about one-half being primiparæ. A large number of the cases were abnormal. Certificates of attendance at this hospital are recognised by all Universities, Colleges, and licensing bodies. Fee for the course of four weeks, £8 8s. Students are accommodated at the new Residential College (5 Cosway Street) opposite the hospital.

Arrangements have also been made for the preliminary instruction in midwifery now required by the General Medical Council. This instruction will include: (1) Practical instruction in the methods of examination of pregnant women; (2) delivery of women in labour under the direct supervision of a medical officer of the hospital; (3) practical instruction in the treatment of the mother and child during the puerperium, including clinics held four times weekly by the visiting medical staff; (4) instruction in the clinical laboratory of the hospital. The fee for this special course for students will be £5 5s. for the course of one month.

### National Hospital for the Paralysed and Epileptic, Queen Square, W.C.

The in- and out-patient practice of this hospital is open at 2 p.m. every day (except Wednesday and Saturday). Patients are seen by the physicians, the physicians for out-patients, and the assistant physicians. Three courses of lectures and clinical demonstrations are given in each year. The fee for out-patient hospital practice and lectures is one guinea for three months; for in-patient clerking a fee of two guineas for three months or three guineas for six months is charged. The museum is available for the prosecution of special work under the pathologist, for which a separate fee of two guineas for the three months is charged.

### Central London Throat and Ear Hospital, Gray's Inn Road, W.C.

Last year 977 in-patients were treated and 11,036 out-patients were seen, involving 48,417 separate attendances. Operation days: In-patients, daily (except Saturday), at 2 p.m.; and out-patients, daily at 9 a.m. (except Saturday). Special courses of practical demonstrations are given weekly on Tuesday and Friday at 3.45 p.m. during the winter and summer sessions by the members of the staff. They are so arranged that practitioners joining at any time are enabled to complete the group of subjects in a course of six weeks. The fee for this course, with daily attendance at the out-patient department, is three guineas. The fee for general clinical attendance is five guineas for three months and eight guineas for six months. All information can be obtained on application to the Dean.

### Hospital for Diseases of the Throat, Golden Square, London, W.

Clinical instruction in the diagnosis and treatment of disease is given daily in the out-patient department from 2 to 5 p.m., on Tuesdays and Fridays from 6.30 to 9 p.m., and on Mondays at 9.30 a.m. The hospital contains 65 beds for in-patients. There is an annual out-patient attendance of over 50,000. Minor operations are performed daily (except Monday) at 9.30 a.m. Major operations are performed on Tuesdays, Wednesdays, Thursdays, Fridays, and Saturdays at 10 A.M. Also Fridays at 2 P.M. Practitioners and medical students are admitted to the practice of the hospital at a fee of five guineas for three months, seven guineas for six months, or ten guineas for perpetual studentship. From amongst the students junior and clinical assistants are appointed periodically. During the winter session the following lectures and demonstrations will be given on Mondays, at 5 P.M. : Dr. Bond, October 13, Carcinoma of the Larynx; Mr. Parker, October 20, Tubercle of the Larynx; Dr. FitzGerald Powell, October 27, Syphilis of the Larynx; Mr. Rose, November 3, Innocent Tumours of the Larynx; Mr. Jefferson Faulder, November 10, Tonsils; Mr. George Badgerow, November 17, Atrophic Rhinitis with Treatment; Mr. Patterson, November 24, Mastoid Complications; Mr. Hope, December 1, Bronchoscopy; Mr. Colledge, December 8, Indications for Mastoid Operations. Lectures on Surgical Anatomy are given on Thursdays at 5 P.M., commencing October 16. Lectures and demonstrations are free to medical practitioners. Apply to George W. Badgerow, F.R.C.S., Hon. Med. Sec.

### The Hospital for Sick Children, Great Ormond Street, W.C.

Clinical instruction is given daily in the wards, out-patient department, and operating theatre by members of the visiting staff. Fees for one month's attendance, two guineas; for three months, five guineas; perpetual ticket, ten guineas; clinical clerk's reduced fee of £1 1s. for three months. Special courses of instruction in the Diseases of Children are held three times during the year. In addition to the ordinary practice of the hospital, special classes illustrated by cases, specimens, skiagrams, operations, etc., are arranged to extend over six weeks of each session. Further details and full syllabus may be obtained by application to the Dean or the Secretary of the hospital. Fee for each course of six lectures £1 1s. Special classes in the "Surgical Diseases of Children" are held on Tuesdays and Fridays from 5.15 to 6.15 P.M. Fee for eight attendances, one guinea. A limited number of clerkships in the pathological department, affording facilities for theoretical and practical instruction in clinical pathology and bacteriology suitable for post-graduates, are available. Fees for one month's course, three guineas; two months' course, five guineas; three months' course, six guineas. A special course of instruction for post-graduates only in the diseases of children of a fortnight's duration will be held from September 29 to October 11 inclusive. For detailed syllabus application should be made to the Dean or the Secretary not later than September 1913. The Dean is Mr. George Waugh, from whom all further information can be obtained, or from the Secretary at the hospital.

### The Hospital for Consumption and Diseases of the Chest, Brompton, S.W.

A course of lectures free to all medical students and practitioners is given three times a year at this hospital. The lectures are given at the hospital at 4.30 P.M. on Wednesdays during term, and are free. Students may enter for courses of clinical instruction in the wards or out-patient department at any time. Arrangements can be made for courses of instruction in special laboratory methods. Special courses of instruction in the diagnosis and treatment of pulmonary tuberculosis are held from time to time. For particulars apply to the Dean.

